# Hypoxia-induced mitochondrial translocation of DNM1L increases mitochondrial fission and triggers mPTP opening in HCC cells via activation of HK2

**DOI:** 10.3892/or.2021.8059

**Published:** 2021-04-19

**Authors:** Min Cai, Ping He, Dian-Liang Fang

Oncol Rep 42: 1125-1132, 2019; DOI: 10.3892/or.2019.7213

Subsequently to the publication of the above paper, the authors have retrospectively realized that they used the human normal liver cell, line L-02, for the experiments reported in this study instead of the intended hepatocellular cell line, Huh-7. Consequently, the results and conclusions reported in this article must be considered to lack reliability.

Therefore, the authors have requested that the article be retracted from the publication. The authors apologize to the Editor and to the readership for any inconvenience caused.

